# Seeing a Face in a Crowd of Emotional Voices: Changes in Perception and Cortisol in Response to Emotional Information across the Senses

**DOI:** 10.3390/brainsci9080176

**Published:** 2019-07-25

**Authors:** Sarah C. Izen, Hannah E. Lapp, Daniel A. Harris, Richard G. Hunter, Vivian M. Ciaramitaro

**Affiliations:** 1 Department of Psychology, Developmental and Brain Sciences, University of Massachusetts Boston, Boston, MA 02125, USA; 2 Division of Epidemiology, Dalla Lana School of Public Health, University of Toronto, Toronto, ON M5T 3M7, Canada

**Keywords:** adaptation, emotion, cortisol, crossmodal, face perception, threat

## Abstract

One source of information we glean from everyday experience, which guides social interaction, is assessing the emotional state of others. Emotional state can be expressed through several modalities: body posture or movements, body odor, touch, facial expression, or the intonation in a voice. Much research has examined emotional processing within one sensory modality or the transfer of emotional processing from one modality to another. Yet, less is known regarding interactions across different modalities when perceiving emotions, despite our common experience of seeing emotion in a face while hearing the corresponding emotion in a voice. Our study examined if visual and auditory emotions of matched valence (congruent) conferred stronger perceptual and physiological effects compared to visual and auditory emotions of unmatched valence (incongruent). We quantified how exposure to emotional faces and/or voices altered perception using psychophysics and how it altered a physiological proxy for stress or arousal using salivary cortisol. While we found no significant advantage of congruent over incongruent emotions, we found that changes in cortisol were associated with perceptual changes. Following exposure to negative emotional content, larger decreases in cortisol, indicative of less stress, correlated with more positive perceptual after-effects, indicative of stronger biases to see neutral faces as happier.

## 1. Introduction

Emotions are expressed and perceived in many different sensory domains, multimodally [1], with emotional information conveyed via faces, voices, odors, touch, and body posture or movement [2,3,4,5]. Our ability to infer the emotional state of others, identify the potential threat they pose, and act accordingly is crucial to social interaction. While more static information conveyed by a face, such as gender or race, can be extracted by visual information alone, more dynamic information, such as emotional state, is often conveyed by a combination of emotional faces and voices. Many studies have examined emotional processing in a given sensory domain, yet, few have considered faces and voices together, a more common experience, which can take advantage of multimodal processes that may allow for more optimal information processing.

### 1.1. Processing Emotion across the Senses

From very early on we can make use of emotional information from multiple sources [6]. For example, infants are able to discriminate emotions by 4 months of age if exposed to stimuli in two different modalities (bimodal), but, only by 5 months of age if exposed to auditory stimuli alone. Likewise, infants are able to recognize emotions by about 5 months if stimuli are bimodal, but not until 7 months if exposed to visual stimuli alone. Starting around 5 months, infants make crossmodal matches between faces and voices [7,8], and by 6.5 months can also make use of body posture information in the absence of face cues [9]. Crossmodal matches also take into account the number of individual faces and voices, with infants, starting at 7 months, showing a looking preference for visual stimuli that match auditory stimuli in numerosity [10]. 

Combining behavioral and event-related potential (ERP) methods, Vogel and colleagues [8] examined the development of the “other-race bias”, the tendency to better discriminate identities of your own race versus identities of a different race. The authors described a perceptual narrowing effect in behavior and brain responses. They found no effect of race on crossmodal emotional matching and no race-modulated congruency effect in neuronal activity in five-month-olds, but found such effects in nine-month-olds, who could only distinguish faces of their own race. Furthermore, seven-month-olds can discriminate between congruent (matching in emotional valence) and incongruent (non-matching in emotional valence) face/voice pairs [7], with a larger negative ERP response to incongruent versus congruent face/voice stimuli and a larger positive ERP response to congruent versus incongruent stimuli. 

These studies in infants, measuring crossmodal matching of emotional stimuli and perceptual advantages in detecting and discriminating emotional information based on bimodal stimulus presentations and the congruency between stimuli, laid important groundwork for the processing of emotional information across the senses. Studies in adults have been more focused on how emotional information in one sense might influence the judgement of emotional information in another sense. To go beyond crossmodal matching or changes in the detection or discrimination of bimodal versus unimodal emotional stimuli, adaptation has been used, mostly in adults, to quantify by how much emotional information in one modality, such as audition, can bias the processing of emotional information in another modality, such as vision. 

### 1.2. Exposure to Emotion: Perceptual Changes 

A powerful tool, adaptation, has been deemed the psychophysicist’s electrode and has been used to reveal the space in which faces are represented. In adaptation, repeated exposure to a stimulus downregulates neuronal firing in response to that stimulus and can yield a perceptual change, a contrastive after-effect. For example, repeated exposure to female faces can bias androgynous faces to appear more masculine [11]. Previous work has shown that many features of a face can be adapted, such as gender, ethnicity, and even emotions (for a review, see Reference [12]).

Adapting to emotional information can bias perception, producing a contrastive after-effect within a sensory modality, either visual or auditory [13,14,15,16]. Repeated exposure to positive faces produces a bias to perceive neutral faces as angry, while repeated exposure to negative faces produces a bias to perceive neutral faces as happy. Complementary biases are found when perceiving neutral sounds after exposure to emotional sounds [13]. Furthermore, the representation of emotion has been shown to be supramodal, with repeated exposure to emotional information in one sensory modality transferring to yield a contrastive after-effect in another sensory modality never directly exposed [17,18,19].

Although emotional information can be adapted within and across the senses, faces and voices often occur simultaneously. Yet, few studies have examined if there is a perceptual advantage to presenting visual and auditory stimuli concurrently and results have been inconclusive. For example, de Gelder and Vroomen [20] found that an emotional voice, happy or sad, could bias perception of a simultaneously presented neutral face to match that of the voice. Similarly, Muller and colleagues [21] found that negative emotional sounds, e.g., screams, could bias perception of a simultaneously presented neutral face to appear more fearful, compared to neutral emotional sounds, e.g., yawns. However, Fox and Barton [22] did not find biased facial perception from emotional sounds. In a related study, using an adaptation paradigm, Wang and colleagues [19] also found no benefit, no increased adaptation, when visual and auditory stimuli were presented together and matched in emotional valence (congruent) compared to when a unimodal visual stimulus was presented in isolation, suggesting that emotional auditory information carried little weight in biasing emotional visual information.

Some discrepancies in results across studies might arise from differences in experimental paradigms. For example, adaptation paradigms may not have been optimized to adapt to emotion per se. Since adaptation effects are stronger when adapting to the same face versus different faces [22] or for easily recognized face/voice pairs, prior studies have often used few exemplar faces and voices. However, if one wants to test for interactions between visual and auditory emotional information, providing many exemplars helps assure one is not adapting to the unique configuration of features of a given face or voice, but rather to emotion. Furthermore, if only a few faces and voices are used and presented as unique pairs during adaptation, presentation of a single stimulus in one modality after adaptation might induce imagery of a stimulus in the other modality due to the associations formed during adaptation. In such a scenario, learned associations induce imagery which then might appear as a strengthening of adaptation effects for stimuli across modalities. In order to promote adaptation to emotion rather than to unique configurations of features of a given face, and to prevent induced imagery of an associated stimulus in the other modality, the current study used 30 unique faces and 15 unique crowd sounds presented at random during adaptation. 

Furthermore, we used crowd sounds, where multiple voices are presented at once, as another way to ensure that unique face/voice pairs did not get formed and to ensure adaptation is to emotion, and not to characteristics of a particular voice or a particular face/voice pair. While many previous studies have used only a few exemplars of face/voice pairs, crowd stimuli can be more informative than single identities. For instance, the gaze of a group is more effective at directing attention than the gaze of an individual [23]. In situations where multiple stimuli are presented at once, it has been shown that participants extract the mean emotion of the stimuli without representing individual characteristics [24,25]. Thus, we expected not only that participants in the current study would efficiently extract the emotional information from multiple voices without representing characteristics of individual voices, but that this information from a crowd would be more informative than information from a single identity.

### 1.3. Exposure to Emotion: Cortisol Changes 

Interestingly, not only can repeated exposure to emotional information alter perception, it can also induce changes in mood, such that exposure to positive emotional content can induce a positive mood and bias perception of faces to be more positive, while induction of a negative mood can bias perception of faces to be more negative (reviewed in Reference [26]). Furthermore, the initial mood of the participant can bias perception, such that a more positive mood at baseline can bias faces to be perceived as more positive [27,28].

In considering how emotional information might alter a physiological marker for the stress response, particularly for negative emotional exposure, we assessed cortisol levels. Cortisol excretion is the final product of hypothalamic–pituitary–adrenocortical (HPA) axis activation in response to stress [29]. Salivary cortisol levels have been used as a non-invasive biomarker of stress response (e.g., [30,31]). Cortisol has also been linked to attention and arousal, with higher cortisol levels correlated with increased attention [32] and have been linked to enhanced emotional face processing [33]. Furthermore, changes in cortisol have been linked to induced negative emotional state such that negative mood induction is associated with elevated cortisol [34,35]. 

Although exposure to emotional information can alter stress levels, the relationship between changes in perception and changes in cortisol following emotional exposure is not well understood. Of note, repeated exposure to emotional information yields opposite effects on perception and mood. While repeated exposure to negative facial emotion biases perception to be more positive (contrastive after-effect), it biases mood to be more negative. It remains to be seen if changes in cortisol positively or negatively correlate with changes in perception. Furthermore, although many studies have investigated the effects of exposure to emotional faces, it is unclear how emotions conveyed by other senses, such as voices, may interact to bias perception and cortisol.

The current study utilized an adaptation paradigm to investigate perceptual shifts, cortisol shifts, and their correlation as a function of exposure to visual and/or auditory emotional information. Participants were exposed to angry faces with or without concurrent emotional sounds that matched (congruent) or did not match (incongruent) facial emotion. We quantified post-adaptation perceptual changes, normalized to baseline perceptual biases, and post-adaptation cortisol changes during the same exposure, normalized to baseline cortisol biases, uniquely for each participant. 

In line with perceptual after-effects, we expected adaptation to negative emotional information would bias perception to be more positive, with stronger effects for congruent versus incongruent emotions. We also assessed perceptual effects post-adaptation to only visual or only auditory emotional information. These conditions provide baseline measures by which to assess differences between congruent and incongruent conditions. Namely, they can distinguish if a congruent emotion enhanced or an incongruent emotion suppressed relative to a baseline measure within a single modality. Given the results of Wang and colleagues [19], we expected the weakest effects following adaptation to only auditory emotional stimuli and expected congruent effects to be stronger and incongruent effects to be weaker than a visual only baseline. 

In line with stress-induced changes in cortisol, we expected exposure to negative emotional information would decrease cortisol if in accord with perceptual effects but increase cortisol if in accord with mood effects. We expected cortisol changes to be largest for congruent emotions and weakest for only auditory emotions. Given pilot data suggesting our negative emotional stimuli were not acutely threatening and not very effective at increasing cortisol, we expected differences in the relative *decrease* in cortisol across adaptation conditions.

## 2. Methods 

### 2.1. Participants

A total of 97 participants, aged 18 or older, were recruited from the University of Massachusetts Boston community and contributed data. Our goal was to gather usable behavioral data in ~20 participants in each of the 4 conditions. A G*power analysis using a medium effect size of 0.5, estimated a total sample size of 73 participants across the 4 conditions of our study. Thus, we aimed to gather data in ~80 useable participants, imagining there would be additional data loss from running cortisol assays. Of our 97 participants, sixteen participants were excluded due to the following: experimenter error (2), biased behavioral responses (4), where the faces that were 80% happy were judged happy less than 75% of the time or faces that were 80% angry were judged happy more than 25% of the time, participant error (3), where participants failed to press the correct buttons to make their responses, or problems with cortisol measurements (7), where cortisol measures were too high or too low relative to normative measures from cortisol standards. Of the 81 participants who contributed usable behavioral data, a subset of 72 contributed usable salivary cortisol samples. 

Our sample consisted of 61 females (mean age = 22.230 years; SD = 3.8747; range = 18–34) and 12 males (mean age = 24.567 years; SD = 11.0601; range = 18–54). One participant did not report their gender and seven did not report their age. Participants reported normal hearing and normal or corrected-to-normal vision, provided written informed consent, and were compensated (US) $20, or received extra credit for an eligible undergraduate course. All experimental procedures and protocols (protocol #2013148) were approved by the University of Massachusetts Boston Institutional Review Board and complied with the Declaration of Helsinki. Please see Table 1 below for detailed demographics.

### 2.2. Questionnaires

Participants completed a demographics questionnaire using the Positive and Negative Affect Schedule–State Version (PANAS). The PANAS uses 20 items to assess the current affective state, separated into negative (NA) and positive (PA) affect subscales, designed to be orthogonal measures [36]. Participants indicated how much they were experiencing each of the listed emotions at that present moment (after we the experimental procedures were described and consent obtained, but before presenting stimuli and starting to adapt) via a 5 point Likert scale. The PANAS subscales for PA and NA were calculated to assess the state-level affect before the study commenced. 

### 2.3. Behavioral Measures

#### 2.3.1. Apparatus 

Visual stimuli were presented on a Nexus cathode-ray tube (CRT) monitor and responses were recorded via laptop keyboard button press using Matlab and the psychophysics toolbox [37,38,39]. Participants were seated 45 cm from the screen and positioned on a chin and forehead rest to maintain stable head position and constant viewing distance. Auditory stimuli were presented via noise-cancelling headphones (3M-Peltor headset), which helped minimize distractions from ambient sounds. 

#### 2.3.2. Stimuli

**Figure 1:**
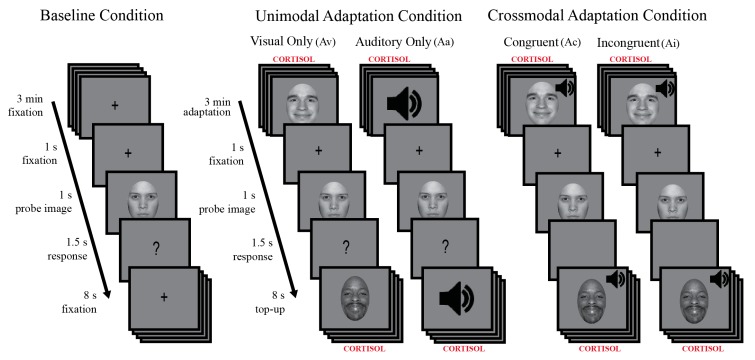
Experimental procedure.

We selected 30 unique angry face images from the NimStim Face Stimulus database [40]. Only faces with validity ratings of 75% or higher for appearing happy or appearing angry were chosen. All stimuli were gray-scaled to 50% and non-facial features, such as hair, were obscured by a grey oval (see sample stimuli in Figure 1, [41]). Overall, 30 possible faces (21 White, 3 Asian, and 6 Black) were presented during the adaptation phase of the task.

A subset of face images was morphed along an emotional continuum. Eight unique identities (4 female and 4 males faces; 5 White, 2 Asian, and 1 Black) were each morphed from a fully affective (100%) happy face to the complementary neutral face for that identity or from a fully affective (100%) angry face to the complementary neutral. We used MorphMan software (version 4.0, STOIK Imaging, Moscow, Russia) to create morphs ranging from neutral to 10%, 20%, 40%, and 80% happy or angry. Each of the 8 identities had 9 morphs (4 angry morphs, 4 happy morphs, and 1 neutral). Overall, 72 possible faces could be presented during the *test phase*.

Participants viewed and judged 64 test faces, presented at random (4 repeats for 80% angry and happy morphs, 8 repeats for 10%, 20%, and 40% angry morphs, happy morphs, and neutral). All faces were 595 × 595 pixels, subtended 19.8^0^ visual angle and were presented at central fixation. Participants were instructed to direct their gaze at central fixation, but eye position was not monitored.

Auditory stimuli were rated by an independent group of 20 UMass Boston undergraduates. Participants listened to 87 one-second sound clips of crowd sounds expressing positive (39) or negative (48) emotions, such as cheering or booing, respectively. Sound clips contained no spoken words, since words might convey different emotional valences for different participants. Sound clips were judged on a 6 point Likert scale for ratings of emotional valence (how angry, how happy, and how mocking) and overall sound quality. Only sound clips with 75% validity ratings for sounding happy or angry and having good sound quality were included. Sound clips conveying mocking emotions were excluded. Overall, 15 positive and 15 negative sounds were presented during the *adaptation phase*. 

#### 2.3.3. Behavioral Procedure

Before the experiment, all participants were familiarized with the procedures. They completed a minimum of 3 practice trials, consisting of an auditory alerting cue, followed by a blank oval (1 s), and then a question mark (1.5 s), during which time participants had to press a button to practice the timing of when to indicate their judgment. For the experiment, each participant completed one baseline and one adapt condition (see Figure 1 below), each lasting 15 min, with a 5 min break between. 

During *baseline*, participants viewed a gray screen with a central fixation cross and were instructed to maintain gaze at central fixation. Following fixation (180 s), a 500 Hz auditory alerting cue was presented briefly followed by 1, out of 72 possible, face morph (1 s). The face morph was followed by a question mark (1.5 s), during which time participants had to judge, via keypress, if the face morph they had just viewed was perceived as happy or angry. Only responses made during the 1.5 s interval were included for analysis, i.e., trials where participants responded too early or too late were not considered incorrect and were excluded. After each response, a grey screen with a fixation cross was presented (8 s). Judgments from a total of 64 face morphs were assessed during baseline and used to determine each participant’s point of subjective equality (PSE), the unique face judged emotionally neutral, equally likely to be perceived as happy or angry (as described below in data analysis for behavioral measures).

*Adaptation* contained the same sequence of events in time as baseline. The crucial difference was that instead of a blank fixation screen for the first 180 s (*initial adaptation*) and for the 8 s (*top-up adaptation*) following each judgment, faces were presented in 1 of 4 possible adaptation conditions. The 4 possible adaptation conditions were (1) **Congruent**: visual face stimuli and auditory crowd sounds matched in emotional valence, such that angry faces were presented concurrently with negative crowd sounds (**Ac**); (2) **Incongruent**: face stimuli and auditory sounds mismatched in emotional valence, such that angry faces were presented with positive crowd sounds (**Ai**); (3) **Visual Alone**: angry faces were presented in isolation, with no concurrent emotional sound (**Av**); and (4) **Auditory Alone**: negative auditory crowd sounds were presented in isolation, with no concurrent emotional face (**Aa**).

During *initial adaptation,* a total of 180 emotional faces (out of 30 unique identities at 100% emotional valence) and/or emotional crowd sounds (out of 15 unique sound clips) were presented (1 s each). During top-up adaptation, 8 emotional faces (100% angry) and/or emotional crowd sounds, were presented (1 s each). Post-adaptation participants judged the same face morphs as in baseline. Judgments from a total of 64 morphed test faces were assessed post-adaptation and used to determine the change in each participant’s unique PSE (as described in the data analysis for behavioral measures below). 

Participants were presented randomly selected faces which had been morphed along an emotional continuum from angry to happy (8 unique face identities: 4 males, 4 females). They judged each face morph as either happy or angry. Baseline started with a 3 min fixation and participants were instructed to maintain central fixation. This was followed by a beep and a face morph presented for 1 s, followed by a 1.5 s response period during which a question mark was presented and participants had to indicate if they thought the previously presented face morph was happy or angry by pressing a key on a keyboard. Baseline consisted of 64 trials. For adaptation, participants were presented the same face morphs and made the same judgments as during baseline. However, adaptation began with a 3 min exposure to 100% angry faces and each judgment was followed by an 8 s top-up exposure during which eight 100% emotional faces were presented, each for 1 s. Adaptation also consisted of 64 trials. Cortisol was collected at the start and 5 min after the end of the adaptation block. A given participant was presented with baseline followed by 1 of 4 possible adaptation conditions. Unimodal adaptation conditions included: Angry faces alone (Av) and negative sounds alone (Aa). Bimodal adaptation conditions included matched emotional valence: angry faces and negative sounds (Ac), and unmatched emotional valence: angry faces and positive sounds (Ai).

#### 2.3.4. Data Analysis for Behavioral Measures

All data was analyzed using Matlab. We used psignifit [42], which implements the maximum-likelihood method described in Reference [43] to fit the data for each participant for the baseline and post-adapt condition separately. We plotted the function such that the *x*-axis represents the emotional morph continuum and the *y*-axis represents the percentage of trials the participant responded that a given face appeared happy. Each participant’s baseline data were fit with a cumulative normal to determine the face morph supporting a 50% happy response, where the subject was equally likely to judge the face as happy or angry. The face judged emotionally neutral was the unique neutral point, or PSE, for the participant and conveys the percent of emotion in a face required to perceive the face as emotionally ambiguous. The PSE is a common measurement used in previous psychophysical studies of crossmodal emotion (for example, References [13,19]). Given our convention of plotting happy emotions to the right of 0 (positive values) and angry emotions to the left of 0 (negative values), a positive baseline PSE indicates a more positive affect is required to see a face as neutral, a negative perceptual bias. Conversely, a negative baseline PSE indicates a more negative affect is required to see a face as neutral, a positive perceptual bias. To quantify the strength of perceptual biases post-adaptation, for each adaptation condition and each participant, we quantified how judgments of the face considered emotionally neutral at baseline changed post-adaptation. Importantly, such a quantification normalizes for any biases existing at baseline. In addition to estimating the PSE as a measure of perceptual bias, we also quantified the slope of the PSE fit, an estimate of variance in the data [44].

**Figure 2:**
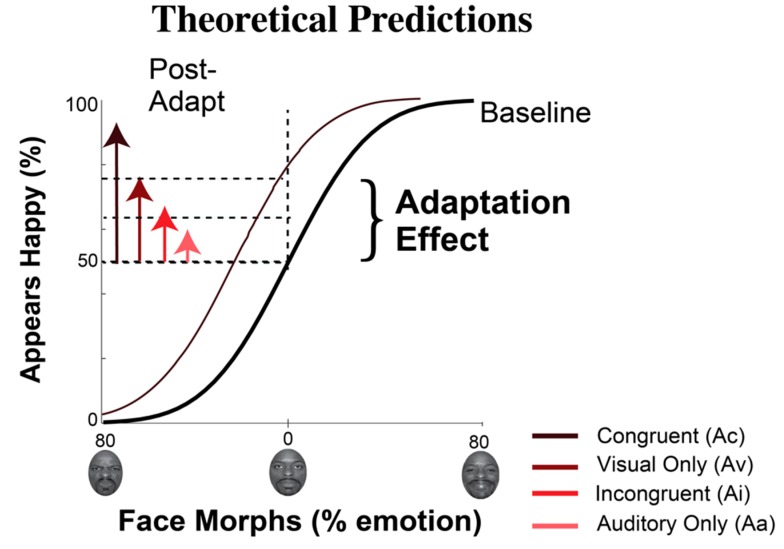
Behavioral predictions.

Figure 2 depicts hypothetical data and fits to illustrate predicted behavioral effects for each adaptation condition. In this hypothetical example, if the face judged neutral at baseline is judged happy on 75% of trials post-adaptation, the shift is a positive bias, as expected from adaptation to negative, angry emotions. 

We expected biases in emotional perception to vary based on adaptation condition. We expected *positive* bias to be (1) strongest after congruent adaptation, where visual and auditory both conveyed *negative* emotional content, (2) moderate after adaptation to only visual *negative* emotional content, (3) weaker after incongruent adaptation, where visual emotions were *negative* but auditory emotions were *positive*, and (4) the weakest after adaptation to only auditory *negative* emotional content. 

The *x*-axis depicts the emotional continuum from 80% angry to 80% happy, with the standard neutral from the NimStim database at zero. The *y*-axis shows the hypothetical percentage of happy judgments. We measured each participant’s unique point of subjective equality (PSE): the face the participant judged equally likely to be happy or angry. The thick black curve reflects a cumulative normal fit to judgments of each face morph at baseline. Baseline PSE was quantified as the face morph supporting 50% happy judgments. The predicted direction and magnitude of changes in the baseline PSE (PSE shift) after adaptation to angry faces are depicted in the direction and magnitude of arrows extending from the baseline PSE. We predicted a positive perceptual bias after adapting to angry faces, a positive PSE shift, with the largest shift for congruent visual and auditory emotional stimuli (Ac), progressively weaker shifts for visual only (Av), and then for incongruent visual and auditory stimuli (Ai), with the weakest shifts for the auditory only condition (Aa). 

### 2.4. Physiological Measures

#### 2.4.1. Quantifying Salivary Cortisol

Saliva samples were collected with salivettes (Sarstedt, Germany), which participants were instructed to chew gently for two minutes. Samples were stored immediately on ice before transfer for longer term storage at −80 °C until quantification. Salivary cortisol levels were quantified using a competitive enzyme-linked immunosorbent assay (ELISA) (Enzo Life Sciences) according to manufacturer instructions. Samples were thawed on ice and centrifuged at 1600× *g* for 10 min at 4°. All samples from a given participant were analyzed on the same plate and samples on the same plate were selected at random from different conditions. 

A micro-plate reader was used to measure optical density at 405 nm. Assay sensitivity was 56.72 pg/mL, intra-assay coefficient of variation was 7.3–10.5%, and inter-assay coefficient of variation was 8.6–13.4%. Specificity was 100% for cortisol and cross reactivity was 3.64% for progesterone and less than 1% for prednisone, testosterone, androstenedione, cortisone, and estradiol. Data from 7 known cortical standards were fit using a 4 parameter logistic curve fitting program (Graphpad PRISM) and concentrations of unknown cortisol samples were determined from this standard curve.

Cortisol samples were collected before (pre-adapt) and starting five minutes after adaptation ended (post-adapt). Given that adaptation lasted 15 min and free salivary cortisol levels should start changing once exposure to stressful stimuli begins and should peak 15–20 min following exposure [45], we should be assessing cortisol changes during or right after our 3 min emotional exposure—the initial adaptation phase. To minimize other factors which could also alter cortisol levels, participants were instructed to refrain from caffeine and exercise for 90 min prior to the experiment and all studies were conducted between 13: 30–18:30 to ensure cortisol levels were not at ceiling and to limit the effects of circadian rhythms on cortisol levels [46,47].

#### 2.4.2. Data Analysis for Cortisol Measures 

Cortisol measurements for each participant were normalized to account for baseline biases, as defined by the equation below:

(Log10(Cortisol post-adapt) − Log10(Cortisol_pre-adapt))/Log10(Cortisol_pre-adapt),(1)

### 2.5. Statistical Analyses

Given that most data were normally distributed, skew/kurtosis between +/−2, parametric statistics were used for data analysis. This was true except for negative affect and cortisol measurements, which were transformed to be normally distributed. A log transformation was applied to cortisol measurements. Outcomes of statistical testing reported reflect tests performed on transformed data where appropriate. 

Planned statistical analyses included ANOVAs for quantifying perceptual shifts and cortisol shifts across adaptation conditions and correlational analyses between perceptual shifts and cortisol shifts. T-tests were used to examine if perceptual and cortisol shifts were significantly different from zero or if there was no change post-adaptation. Non-parametric tests were used to assess changes in slope across adaptation conditions. We also examined baseline biases that could potentially minimize the size of perceptual or physiological shifts following adaptation. Planned analyses included correlations between baseline biases in perception (PSE) and cortisol, perception, and mood (PANAS-PA and NA) and cortisol and mood. 

## 3. Results

### 3.1. Behavioral Measures

Of the 64 possible test trials of face morphs used to determine perceptual biases in judging emotion, participants completed an average of 57.49 (SD = 7.01) baseline test trials and 60.21 (SD = 5.43) post-adaptation test trials. 

In order to assess the goodness-of-fit of the psychometric function to our data, we considered measures of deviance, quantified using psignifit, for PSE measures at baseline and post-adaptation. On average, the deviance at baseline was 5.50 (SD = 2.82) and post-adaptation was 5.33 (SD = 2.89).

An ANOVA, Bonferroni corrected to account for multiple comparisons (alpha = 0.0083), was run to test the hypothesis that the strength of changes in perception varied across adaptation conditions. We expected the largest perceptual change, shift in PSE, for congruent adaptation, an intermediate effect for only visual adaptation, relatively weaker changes for incongruent adaptation, and the weakest change for only auditory adaptation. 

**Figure 3:**
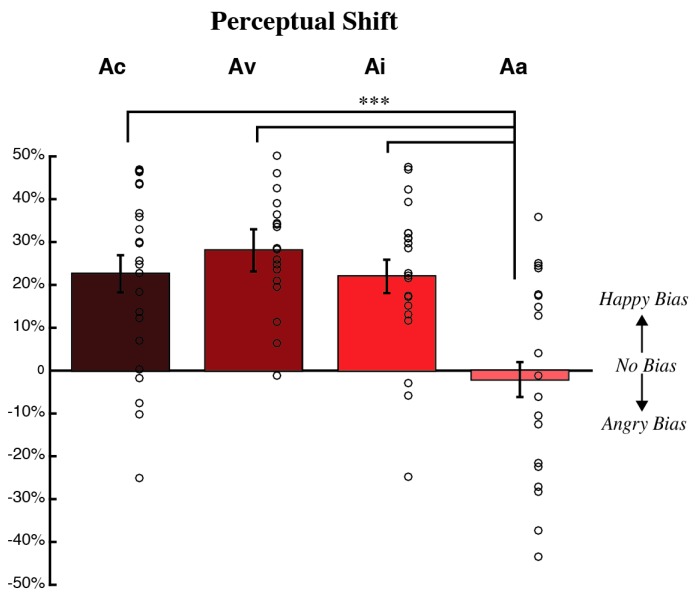
PSE shifts, the change in judgments of the face judged neutral at baseline after adaptation, are shown for each adaptation condition. The *x*-axis depicts the adaptation condition and the *y*-axis depicts the mean PSE shift, normalized by baseline (+/− SEM across participants), with data from individual participants shown via open circles. A mean shift in the negative direction indicates that the unique face judged neutral at baseline looked angrier, on average, after adaptation. Conversely, a mean shift in the positive direction indicates that the unique neutral face looked happier, on average, after adaptation. After adapt angry, the PSE shift was significantly more positive for the congruent, visual only, and incongruent conditions compared to the auditory only condition (*** indicates *p* ≤ 0.001). Furthermore, all adapt angry conditions, except auditory only, showed a significant positive PSE shift.

We found a significant main effect of adaptation condition on PSE shift after adapting to negative emotions (*F*(3,77) = 9.080, *p* < 0.001, partial *η^2^* = 0.261; see Figure 3, with a significantly more positive PSE shift for Ac, Ai, and Av compared to Aa. This indicated that the mean neutral face appeared happier post-adaptation for Ac, Ai, and Av relative to Aa (Ac: *t*(40) = 3.584, *p* < 0.001; Av: *t*(35) = 4.716, *p* < 0.001; Ai: *t*(38) = 3.657, *p* = 0.001). No significant differences were found between other conditions (Ac versus Av: *t*(39) = −0.964, *p* = 1; Av versus Ai: t(37): 1.183, *p* = 1; Ac versus Ai: t(42) = 0.100, *p* = 1). One sample *t*-test indicated all conditions except Aa showed significant adaptation effects; PSE shifts were significantly different from baseline (Ac: *t*(22) = 5.185, *p* < 0.001; Av: *t*(17) = 8.833, *p* < 0.001; Ai: *t*(20) = 5.674, *p* < 0.001; Aa: *t*(18) = −0.384, *p* = 0.706). 

We also examined perceptual shifts with a Bayesian ANOVA in JASP (JASP Team, 2018), using a Cauchy prior distribution with *r* = 1/sqrt(2). The Bayes factor (BF_10_) of 654.274 suggests that the data were approximately 650 times more likely to occur under the alternative hypothesis than under the null (suggesting extreme evidence), with an error percentage of 0.011%. This indicates that PSE shift differs as a function of adaptation condition. Post-hoc tests corrected for multiple testing by fixing the prior to 0.5 suggest a similar pattern of results to the ANOVA above: more positive PSE shifts for Ac, Ai, and Av compared to Aa (Ac: posterior odds = 13.946, Ai: posterior odds = 16.031, Av: posterior odds = 206.343) and no differences between other conditions (Ac versus Av: posterior odds = 0.184; Av versus Ai: posterior odds = 0.224; Ac versus Ai: posterior odds = 0.124). Bayesian one sample *t*-tests indicated all conditions, except Aa showed an adaptation effect (Ac: BF_10_ = 724.4, error < 0.001%; Av: BF_10_ = 157,852, error < 0.001%; Ai: BF_10_ = 1531, error < 0.001%; Aa: BF_10_ = 0.254, error < 0.05%).

Although PSE shifts did not differ across congruent, incongruent, and visual only conditions, another quantification of our data, slope, might differ, with steeper slopes indicative of less variance in perceptual data [44]. However, we found no significant differences in slope changes across adaptation conditions (*p* = 0.919; data not shown). 

### 3.2. Physiological Measures 

An ANOVA, Bonferroni corrected to account for multiple comparisons (alpha = 0.0083), was run to test the hypothesis that the strength of changes in cortisol varied across adaptation conditions. We expected the largest cortisol change for congruent adaptation, an intermediate change for only visual adaptation, relatively weaker changes for incongruent adaptation, and the weakest change for only auditory adaptation. Furthermore, we expected relatively weak increases in cortisol and mostly effects on relative differences in decreases in cortisol.

**Figure 4:**
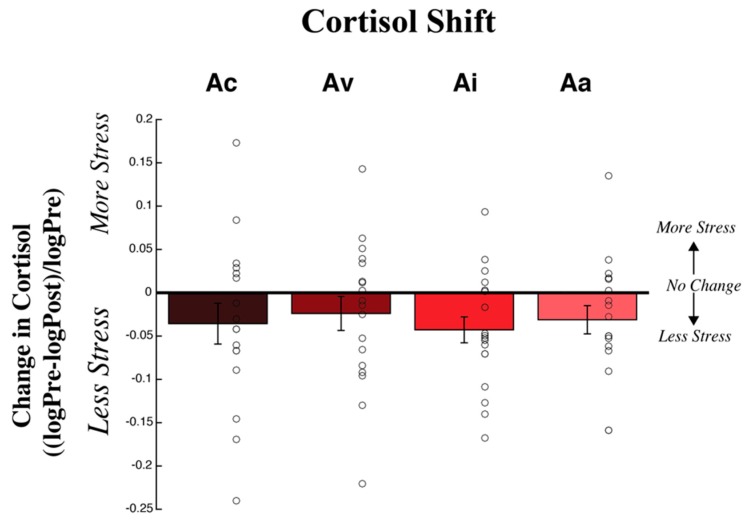
Cortisol Shifts.

We found no significant main effect of adaptation condition on cortisol shift after exposure to negative emotions (*F*(3,58) = 1.618, *p* = 0.195, partial *η^2^* = 0.077; see Figure 4. One sample *t*-test indicated cortisol changes were significantly different from baseline, after exposure to negative emotions, except for condition Aa (Ac: *t*(12) = −2.562, *p* = 0.025; Av: *t*(16) = −3.184, *p* = 0.006; Ai: *t*(13) = −5.224, *p* < 0.001; Aa: *t*(17) = −2.027, *p* = 0.059).

We also examined physiological shifts with a Bayesian ANOVA in JASP (JASP Team, 2018), using a Cauchy prior distribution with *r* = 1/sqrt(2). The Bayes factor (BF_01_) of 2.314 suggests that the data were approximately 2.3 times more likely to occur under the null hypothesis than under the alternative, with an error percentage of < 0.001%. This indicates that the strength of cortisol shifts did not vary under different adaptation conditions. Bayesian one sample *t*-tests indicated cortisol changes were more likely to occur under the alternative relative to the null hypothesis for all conditions, with varying degrees of evidence (Ac: BF_10_ = 2.779, error < 0.005%; Av: BF_10_ = 8.505, error < 0.001%; Ai: BF_10_ = 186.5, error < 0.001%; Aa: BF_10_ = 1.276, error < 0.01%).

Changes in cortisol levels are shown for each adaptation condition. The *x*-axis depicts the adaptation condition and the *y*-axis depicts the mean change in cortisol, normalized by baseline (+/− SEM across participants), with data from individual participants shown via open circles. A mean shift in the negative direction indicates that cortisol decreased post-adapt relative to pre-adapt. Conversely, a mean shift in the positive direction indicates that cortisol increased post-adapt relative to pre-adapt. There were no significant differences in cortisol shift across conditions and mean cortisol shifts tended to be negative.

### 3.3. Correlations between Behavioral and Physiological Measures

**Figure 5:**
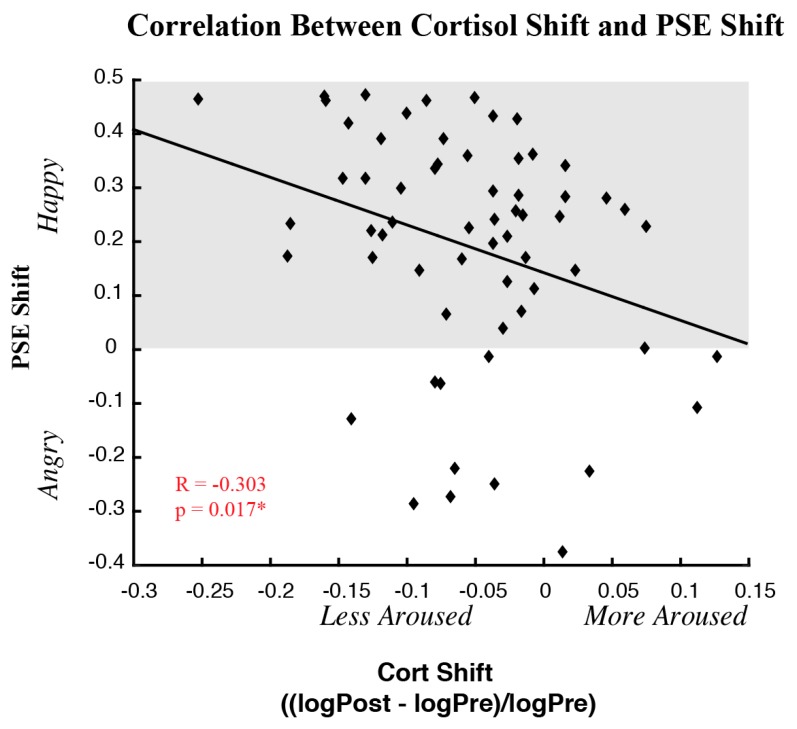
Correlations between cortisol and PSE shifts. Correlations between cortisol shifts and PSE shift across participants are shown collapsed across adapt conditions. The *x*-axis depicts cortisol shift, with positive values indicating an increase and negative values a decrease in cortisol levels post-adapt relative to pre-adapt. The *y*-axis depicts PSE shift, with positive values indicating an increase in happy judgements and negative values indicating an increase in angry judgements, relative to baseline. There was a significant negative correlation between cortisol and PSE shift across adapt conditions (*r* = −0.303; *p* = 0.017) (* indicates *p* ≤ 0.05).

Given the high variability in our data, considering only differences in means across participants might obscure important relationships arising from individual differences. Thus, we tested whether shifts in the perception of emotion (PSE shift) correlated with shifts in cortisol, using a Pearson correlation between PSE and cortisol shifts (see Figure 5). We found a significant negative correlation between PSE shifts and cortisol shifts across adapt conditions (*r* = −0.303, *p* = 0.017). This negative correlation suggests that, following exposure to negative emotions, as the neutral face was judged more positive, post-adapt cortisol levels were more negative relative to baseline cortisol levels, possibly due to lower stress, lower arousal, or less attention. The same correlation run with Bayesian statistics yielded a BF_10_ of 2.596, suggestive of anecdotal evidence for a relationship between shifts in cortisol and shifts in perception. 

### 3.4. Underlying Biases in Behavioral and Physiological Measures

All our measures quantifying perceptual and cortisol changes were normalized to starting baseline values. We examined perceptual and cortisol measures at baseline, to determine if baseline biases could influence the effects of interest. We found no significant main effect of adaptation condition on perception at baseline nor on cortisol at baseline (perception: *F*(3,77) = 0.803, *p* = 0.496, partial *η^2^* = 0.030; cortisol: *F*(3,58) = 0.506, *p* = 0.680, partial *η^2^* = 0.026; data not shown) and no significant correlations between baseline cortisol and baseline PSE (*r* = 0.0180; *p* = 0.161; data not shown), or baseline PSE and baseline state affect (PA: (*r* = 0.196; *p* = 0.192); NA: (*r* = 0.055; *p* = 0.647; data not shown)). 

Of note, while not a main measure, as state affect was only assessed before but not after adaptation, we found a significant negative correlation between cortisol and positive affect at baseline (*r* = −0.237, *p =* 0.037; data not shown), such that more elevated cortisol at baseline was associated with less positive affect at baseline. No significant correlation was found between cortisol and negative affect at baseline (*r* = −0.117, *p* = 0.369; data not shown).

## 4. Discussion

We assessed the relative contributions of visual and auditory emotional information in biasing changes in perception and cortisol and the correlation between the strength of changes in perception and changes in cortisol. Unlike previous work using unique face-voice pairings for only a few individual face identities, we used a wide range of facial identities and unassociated emotional crowd sounds to assess emotional processing.

We hypothesized that (1) the emotion perceived in a face would show a positive bias post-exposure to negative emotional information, in accord with contrastive perceptual after-effects, and that (2) such after-effects would vary based on whether emotion was conveyed by visual and/or auditory information and whether visual and auditory emotional valence matched. Overall, we found exposure to negative emotions yielded positive perceptual biases (positive PSE shift) in all but the auditory only adaptation condition, which showed no effect. In accord with our expectations and replicating previous literature, PSE shifts were weakest following only auditory emotional exposure. Contrary to our expectations, the magnitude of PSE shifts did not differ for congruent versus incongruent emotions nor for congruent versus visual only emotions. The failure to find a benefit for congruent versus visual only adaptation was also noted in a previous study using unique face-voice pairings and finding no benefit, no increased PSE shift, for congruent visual and auditory happy emotions versus only visual happy emotions [19]. 

We hypothesized that cortisol would decrease after exposure to negative emotional information and that decreases would vary based on whether the emotion was conveyed by visual or auditory information and whether visual and auditory emotional valence matched. Overall, we found cortisol decreased after exposure to negative emotional information, but we found no significant differences as a function of adaptation condition. 

Given the variability of perceptual and cortisol shifts across individuals, we also tested the correlation between the magnitude of perceptual shifts and cortisol shifts. Here, we found a significant negative correlation across participants, such that the stronger the positive bias in perceiving a face after exposure to negative emotional content the stronger the decrease in cortisol. While perceptual shifts correlated with cortisol shifts, baseline cortisol levels did not correlate with baseline perceptual biases. Thus, underlying baseline differences could not account for the correlations we observed between shifts in perception and shifts in cortisol.

### Is Cortisol a Proxy for Stress, Arousal or Attention? 

Our results highlight that changes in cortisol may correlate with changes in perception: the more that exposure to angry emotions biases faces to be perceived as happy the *more pronounced* the *decrease* in cortisol. This is contrary to what one might expect if changes in cortisol correlated with changes in mood. Thus, while repeated exposure to negative emotional content increases *positive* biases in perception, such exposure increases *negative* biases in mood. One would expect negative biases in mood to correlate with a *less pronounced* decrease in cortisol, or even an increase in cortisol. Yet, we find that exposure to negative emotional content yielded larger, not smaller, *decreases in* cortisol such that the larger the decrease in cortisol the greater the *increase in positive* perceptual bias. Thus, in our paradigm and with our emotional stimuli, changes in cortisol correlate with changes in perception rather than changes in mood. 

One might have expected *changes* in cortisol to serve as a proxy for *changes* in mood since some studies find a correlation between baseline mood and baseline cortisol levels. For example, some studies find that positive affect correlates with decreased cortisol and negative affect with increased cortisol levels [48,49]. Yet, while several studies find urinary and salivary cortisol levels are associated with anxiety and depression [50,51], or with negative state affect [34,48], other studies do not find an association between negative trait affect and cortisol level [51,52]. Taken together, these studies do not provide a clear picture of the relationship between positive or negative state affect and cortisol levels. 

Of note, although cortisol has long been known as the stress hormone, it has also been referred to as a marker of attention and arousal. Van Honk and colleagues [32] found that baseline cortisol levels correlated with the probability of orienting away from threatening faces, suggesting that individuals with higher baseline cortisol levels had higher levels of arousal, or more engaged attention, and thus responded more quickly to threatening faces than individuals with lower cortisol levels. More recently, Kagan [53] highlighted that the term “stress” is assigned too broadly and should be reserved for describing reactions to an experience that directly threatens an organism. He describes cortisol as a marker for exploratory activity and responses to novel situations. In response to Kagan [53] and McEwen and McEwen [54] call for more investigations into epigenetic factors that might underlie cortisol responses to positive and negative events to help clarify distinctions between “good stress”, “tolerable stress”, and “toxic stress”. They emphasize that responses to stressors are highly individualized and that early life stressors may result in a reduced ability to cope in certain stressful situations.

## 5. Limitations

It is possible that certain aspects of our experimental design could have minimized the shifts in perception and/or cortisol we observed. For example, given that directing attention to emotional information can enhance adaptation effects (e.g., [55]) and given the possibility that changes in cortisol may reflect changes in attention state, the failure to optimize attention to emotional information in the current study could have minimized the shifts we observed in perception and/or cortisol. Individual differences in attending more to visual or auditory emotional information could also have influenced results.

Furthermore, the stimuli used in this study could have yielded weaker shifts in perception and/or cortisol. We expected smaller adaptation effects given our use of multiple face identities (e.g., [22]) and no unique face-voice pairings. Yet, it was important to use such stimuli to minimize the confounds of adapting to unique face or voice features and to minimize visual imagery in assessing the transfer of emotion across the senses. While a review is beyond the scope of this paper, studies have begun to consider behavioral as well as neural correlates of crossmodal emotional processing yet have found conflicting results (for reviews, see [56,57]). For example, while some ERP studies find enhanced behavioral and neuronal processing for congruent bimodal compared to unimodal emotional stimuli [58], other studies find neuronal but not behavioral enhancement [59,60]. 

In our study, we also did not evaluate social anxiety status. Yet, social anxiety status can affect the physiological stress response, such that basal cortisol levels are higher in individuals high in social anxiety, and HPA axis reactivity may be blunted, yielding weaker changes in cortisol in those high in social anxiety [61]. If some individuals in our sample were high in social anxiety, this could have obscured and/or minimized the changes in cortisol we observed. Further study of crossmodal emotional processing in general, and in social anxiety in particular, is warranted (e.g., [62]). 

Finally, we also could not assess the effect of race on emotion perception. Perceptual narrowing early in life creates more salient face representations for faces of one’s own race relative to those of another race [63]. Thus, identity discrimination is easier for faces from one’s own race compared to faces from another race. This has been called the other-race effect, or other-race bias (see Reference [64] for a review). Further, studies in both infants and adults suggest that emotional perception is more accurate when viewing a face of the participants’ same race versus a different race [8]. The current study did not have a large enough sample size to consider how the race of the participant could have influenced perceptual processing of emotional information for faces of one’s own race versus a different race. Furthermore we only sampled a limited number of races in the faces presented. Thus, we did not have adequate power to examine this effect, and cannot come to any meaningful conclusion about the effect of race in this context. Future research should assess the role of race in crossmodal emotional perception.

## 6. Conclusions

We studied crossmodal emotional processing to quantify if perceptual and physiological effects were stronger if visual and auditory emotions were matched in valence (congruent) or unmatched (incongruent). We quantified how emotional exposure altered perception, using psychophysics, and how it altered a physiological proxy for stress or arousal using salivary cortisol. This is an interesting question as repeated exposure to emotional content can induce a contrastive perceptual after-effect in the opposite direction (i.e., adapting negative emotion induces a positive emotional bias) but also induce mood in the same direction, making it unclear how changes in cortisol may be related. The results of our study suggest a relationship between perceptual changes and changes in a physiological measure of stress, cortisol, such that after exposure to negative emotional face content, larger decreases in cortisol correlated with more positive perceptual after-effects. This suggests an orthogonal relationship between measurements of stress and perceptual after-effect, with adaptation to negative emotional face stimuli inducing both a bias to see neutral faces as happier, and a decrease in stress as measured by cortisol. Additionally, while we observed a perceptual bias to judge faces as happier in all 3 conditions of adaptation to negative faces (congruent, incongruent, and visual only), we did not observe significant differences in adaptation strength across conditions except for all three of the above conditions differing from an auditory only condition, which failed to show a significant perceptual aftereffect. We also found no significant differences in cortisol changes across our 4 adaptation conditions.

## Figures and Tables

**Table 1:** Demographics.

Demographics		Congruent	Incongruent	Visual	Auditory
**Mean Age (SD)**		20.5 (2.2)	22.5 (4.7)	25.5 (9.5)	22.1 (2.8)
**White**					
**Male**	*N*	0 (0%)	1 (50%)	2 (33.3%)	0 (0%)
**Female**	*N*	7 (36.8%)	7 (36.8%)	6 (50%)	8 (50%)
**Hispanic**					
**Male**	*N*	1 (25%)	0 (0%)	2 (33.3%)	1 (33.3%)
**Female**	*N*	4 (21.1%)	7 (38.9%)	2 (16.7%)	0 (0%)
**African/African American**					
**Male**	*N*	0 (0%)	0 (0%)	1 (16.7%)	0 (0%)
**Female**	*N*	1 (5.3%)	0 (0%)	3 (25%)	4 (25%)
**Asian**					
**Male**	*N*	0 (0%)	1 (50%)	0 (0%)	1 (33.3%)
**Female**	*N*				
**Multiracial**					
**Male**	*N*	0 (0%)	0 (0%)	0 (0%)	0 (0%)
**Female**	*N*	1 (5.3%)	0 (0%)	0 (0%)	1 (6.3%)
**Unspecified**					
**Male**	*N*	3 (75%)	0 (0%)	1 (16.7%)	1 (33.3%)
**Female**	*N*	3 (15.8%)	2 (11.1%)	0 (0%)	0 (0%)
